# Does Minimally Invasive Approach Change Criteria of Allocation to Treatment Strategy in Synchronous Colorectal Metastases? An Italian National Registry-Based Analysis

**DOI:** 10.3390/cancers18030479

**Published:** 2026-01-31

**Authors:** Giorgio Traina, Alessandro Ferrero, Felice Giuliante, Andrea Ruzzenente, Giorgio Ercolani, Umberto Cillo, Vincenzo Mazzaferro, Giuseppe Maria Ettorre, Andrea Belli, Elio Jovine, Rebecca Marino, Pierpaolo Sileri, Francesca Ratti

**Affiliations:** 1 Hepatobiliary Surgery Division, IRCCS San Raffaele Scientific Institute, 20134 Milan, Italy; traina.giorgio@hsr.it (G.T.);; 2 Department of Surgery, Mauriziano Hospital, 10128 Turin, Italy; 3 Hepatobiliary Surgery Unit, Fondazione Policlinico Universitario A. Gemelli IRCSS, Università Cattolica del Sacro Cuore, 00136 Rome, Italy; 4 Division of General and Hepatobiliary Surgery, G.B. Rossi University Hospital, University of Verona, 37134 Verona, Italy; 5 General and Oncology Surgery, Morgagni-Pierantoni Hospital, Ausl Romagna, 47121 Forlì, Italy; 6 Department of Medical and Surgical Sciences, Alma Mater Studiorum, University of Bologna, 40138 Bologna, Italy; 7 Hepato-Biliary-Pancreatic Surgery and Liver Transplant Unit, General Surgery 2, Department of Surgical, Oncological and Gastroenterological Sciences, University of Padua, 35128 Padua, Italy; 8 Department of Surgery, Division of HPB Surgery and Liver Transplantation, Fondazione IRCCS Istituto Nazionale Tumori Di Milano, 20133 Milan, Italy; 9 Department of Oncology and Hemato-Oncology, University of Milan, 20122 Milan, Italy; 10 Department of Surgery, San Camillo Forlanini Hospital, 00152 Rome, Italy; 11 Hepatobiliary Unit, Division of Surgical Oncology, Istituto Nazionale Tumori IRCCS Fondazione “G. Pascale”, 80131 Naples, Italy; 12 Department of General Surgery, IRCCS Azienda Ospedaliero-Universitaria Di Bologna, 40138 Bologna, Italy; 13 Colorectal Surgery Division, IRCCS San Raffaele Scientific Institute, 20134 Milan, Italy; 14 Faculty of Medicine and Surgery, Vita-Salute San Raffaele University, 20134 Milan, Italy

**Keywords:** minimally invasive, laparoscopic, robotic, colorectal liver metastases, synchronous

## Abstract

**Background/Objectives**: Heterogeneity in clinical scenarios of colorectal liver metastases (CRLM) leads to the possible application of different surgical strategies. Specifically, the possibility of performing combined colorectal and liver resections for synchronous CRLM has been proposed in specific settings but its feasibility, safety and impact in minimally invasive settings remain underexplored. This study examines a multicenter Italian experience, comparing perioperative outcomes of combined (CR) versus non-combined (NCR) minimally invasive liver resections (MILR) for CRLM. **Methods**: Patients from the prospective multicenter registry of the Italian Group of Minimally Invasive Liver Surgery (I Go MILS) who underwent MILR for CRLM between 2016 and 2024 were included. Perioperative outcomes were compared between CR and NCR using Nearest Neighbor Matching. **Results**: In total, 2286 patients were analyzed, including 1879 NCR and 407 CR. CR was associated with less challenging resections (technical difficulty Kawaguchi grade III: 7.13% vs. 14.53%, *p* < 0.001), longer operative time (385 vs. 270 min, *p* < 0.001) and higher major complication rate (11.55% vs. 5.11%, *p* < 0.001) compared to NCR. The conversion rate was similar between the two groups (9.09% vs. 7.91%, *p* = 0.479). Technical complexity, operative time, conversion, low-volume hospital, and CR was an independent predictor of major complications after matching. **Conclusions**: CR is associated with a higher risk of postoperative complications, despite being selected for minor liver resections, confirming the impact of associated colorectal surgery in determining the postoperative risk and hence highlighting the concept that accurate preoperative patient selection is a key step in guiding treatment allocation for CRLM. Therefore, MILR does not yet justify broadening indications for combined resection beyond carefully selected patients.

## Simple Summary

This multicenter Italian study compared minimally invasive liver resections with combined minimally invasive colorectal/liver resections for synchronous colorectal liver metastases. The addition of the colorectal procedure increased postoperative morbidity, even though conversion rates remained similar. Combined minimally invasive resections are feasible but should be limited to carefully selected patients, ideally treated in high-volume centers. Overall, minimally invasive techniques reduce some risks but do not offset the added morbidity of performing both colorectal and liver resections simultaneously, supporting the need for strict patient selection and further research on long-term outcomes.

## 1. Introduction

Colorectal cancer ranks as the third most frequent cancer and the second leading cause of cancer-related mortality worldwide [1]. Between 20 and 25% of patients present with metastatic disease at diagnosis, with metastases being the primary cause of death [2] and synchronous presentation being one of the main determinants of long-term prognosis. Surgical resection with R0 margins is potentially curative [3], but the superiority of one of the three available strategies (i.e., liver resection first, colic resection first and combined resection) has not been clearly demonstrated [4,5,6,7,8,9,10,11,12,13,14,15,16,17]. International guidelines [18,19] recommend a bowel-first approach if the tumor is symptomatic or with high risk of colic obstruction or perforation, while a liver-first strategy should be considered in patients with high burden of liver disease—to minimize the risk of progression to unresectability—or in rectal cancers to allow the period of chemo-radiotherapy while waiting for reassessment of the rectal primary tumor. A combined approach is reserved for selected patients with low burden of liver disease, requiring a minor hepatic resection. In cases of combined resection (CR), the entire tumor burden can be removed in a single operation, reducing the number of hospitalizations but potentially increasing surgical complexity [15,20,21,22].

With the widespread adoption of the Minimally Invasive (MI) approach—both robotic and laparoscopic—in the world of hepatic surgery (Minimally Invasive Liver Surgery, MILS), few reports have been published documenting the benefits of minimally invasive techniques in the setting of CR, leading to a reduction in morbidity rate and length of stay compared with the open counterpart [14,23,24,25,26,27].

While advantages of minimally invasive techniques have been widely documented—through randomized controlled trials and meta-analyses—in the setting of isolated resections for metastases [14,24,25], the impact of adding a minimally invasive colorectal resection compared to hepatic resection alone has been poorly investigated: nevertheless, the benefit conferred by the possibility of adopting minimally invasive techniques in CR could influence therapeutic decisions in the algorithm for allocation to the combined or staged strategy.

The primary endpoint of this study was to evaluate—within the Italian prospective Registry on MILS (i.e., I Go MILS Registry)—the impact of concomitant colorectal surgery in patients submitted to MILS surgery (either laparoscopic or robotic) for liver metastases from colorectal cancer (CRLM) in the setting of synchronous CRLM comparing this group of patients with those undergoing MIS surgery for CRLM without concomitant colorectal surgery. The impact of colorectal surgery was specifically evaluated in terms of MIS feasibility (i.e., conversion rate) and short-term morbidity (either liver-specific or colon-specific). The secondary endpoint was to evaluate the criteria of allocation to combined resections in the current national scenario.

## 2. Materials and Methods

### 2.1. Study Design

All consecutive patients undergoing MILS for CRLM between 2016 and the first trimester of 2024 and prospectively enrolled in the I Go MILS Registry were included and analyzed retrospectively for the purposes of the present study.

Patients were divided into two groups according to treatment strategy: the Combined Resection Group (CR Group) included patients who underwent combined minimally invasive colic/rectal and hepatic resection, while the Non-Combined Resection Group (NCR Group) included patients who underwent minimally invasive liver resections. Propensity score matching was adopted to reduce confounding factors and conduct the primary endpoint analysis, i.e., outcome comparison and feasibility evaluation. An analysis including the unmatched cohorts was performed to evaluate the criteria of allocation to combined resections, hence addressing the secondary endpoint. No particular exclusion criteria were utilized to select patients from the I Go MILS Registry.

### 2.2. The I Go MILS Registry

The Italian Group of Minimally Invasive Liver Surgery (I Go MILS) is the prospective registry established in Italy to create a national database to monitor, track the trends and promote the diffusion of minimally invasive surgery in the country. The Registry is prospective and any Italian center performing MILS is allowed to join without restriction criteria based on annual caseload of activity in liver surgery or MILS [28,29], so both peripheral general surgery departments and expert hepatobiliary units participate in this registry.

Candidates for MILS are consecutive and enrolled within an intention-to-treat perspective: the informed consent form—where candidates can read about the study protocol and data protection—must be signed before enrollment.

Data of enrolled patients are collected and stored in an electronic case report form (eCRF-web based) with log-in allowed through ID and password provided only to the authorized personnel. Each patient is identified by a code which is automatically generated by software once the patient is enrolled. The data are then processed and disseminated in an anonymous form.

The case report form is composed of three separate modules: enrollment, surgery and postoperative data. The total number of variables required to complete the CRF is 37. A detailed description of the dataset is reported elsewhere [29].

### 2.3. Variables

The following variables were collected from the registry: demographic characteristics (age, sex, BMI), liver-specific data (portal hypertension, hepatic parenchyma status, tumor dimension), surgical information (type of hepatic resection, technical complexity, type of minimally invasive approach, operative time, blood loss, intraoperative blood transfusions, Pringle maneuver, conversion to laparotomy and reason for conversion), postoperative outcomes (length of stay, complications, mortality), volume of activity of the center where the patient was treated.

Operative mortality was defined as death within 90 days after surgery. Morbidity was graded according to the Dindo–Clavien classification [30]; a complication classified with a Dindo-Clavien of at least 3a was considered severe.

### 2.4. Stratification of Centers and Complexity

For the purposes of the present study, the center case volume was defined as the number of MILS for CLRM performed per year, calculated as the overall number of procedures undertaken by a center divided by the number of years of enrollment in the registry. The cut-off to identify high-volume centers was 10 procedures per year.

Kawaguchi et al.’s [31] classification was adopted to classify and stratify each hepatic resection in three groups of technical difficulty. This scoring system was created to classify laparoscopic liver resections according to operative time, intraoperative blood loss, conversion rate, morbidity and mortality. Briefly, this classification consists of three difficulty groups. The first group includes wedge resections and left lateral sectionectomy, the second group includes left hepatectomy and right anterior sectionectomy, while the third group includes right posterior sectionectomy, right hepatectomy, central hepatectomy and right/left trisectionectomy. This classification is internationally accepted and validated and could be promptly adopted in the setting of the I Go MILS Registry.

### 2.5. Statistics

Descriptive variables are expressed as median and interquartile ranges, while the categorical variables are presented with proportions and percentages. Categorical variables were compared using the chi-squared test or Fisher’s exact test, as appropriate. Continuous variables were analyzed using the Mann–Whitney U test.

To minimize imbalances between the groups, a propensity analysis with a Nearest Neighbor Matching algorithm was used in a 3:1 ratio, as the two groups are numerically unbalanced. The variables included in the model were age, body mass index (BMI), gender, high-volume center, characteristics of underlying liver parenchyma, lesion dimension (>30 mm) and technical difficulty grade. The caliper was set equal to 0.020, and matching was performed without replacement [32,33].

Univariate and multivariate logistic regression analyses for predictors of severe postoperative complications were conducted before and after the Nearest Neighbor Matching. Odds ratios (ORs) with 95% confidence intervals (CIs) and *p*-values were calculated for each predictor to assess their association with severe postoperative complications. *p* < 0.050 was considered statistically significant for all tests. Stata^®^ version 14 (StataCorp, College Station, TX, USA) was used for all analyses.

## 3. Results

### 3.1. Time Trend of Enrollments

**Figure 1:**
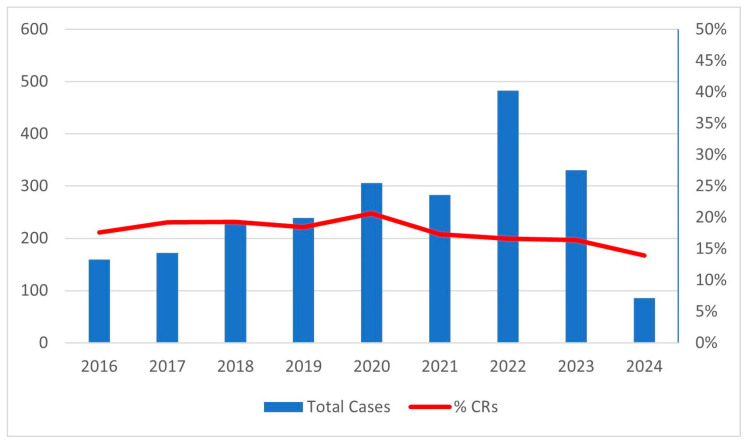
Number of cases enrolled each year and percentage of CRs each year.

During the study period, 2286 patients with CRLM were enrolled in the I Go MILS Registry from 38 Italian centers, 11 of which were considered high volume with a mean of 20.1 MILS for CRLM/year. In total, 1879 patients were included in the NCR group and 407 in the CR group. The number of combined resections over the total number of MILS resections remained substantially unchanged throughout the recruitment period, while the number of patients included each year progressively increased, from almost 150 patients/year at the beginning to over 300 patients/year in the last years (Figure 1).

### 3.2. Comparison Between CR and NCR Groups Before Matching

The baseline characteristics of patients in the two groups are provided in Table 1. The median age was 67 years, the median BMI was 25 kg/m2, and there were 58% and 61% male patients, respectively, in the CR and NCR groups. No significant differences between the groups were found for these variables, as well as for the conditions of underlying liver parenchyma.

Differences between groups were detected regarding the dimensions of the hepatic largest lesion: in the NCR cohort, 39.2% of the lesions were larger than 3 cm compared to 30.5% in the CR cohort (*p* < 0.001).

Regarding surgical characteristics (Table 1 and Table 2), wedge resections were more frequently performed in the CR group (54.3% vs. 34.9%), while other types of hepatic resections were performed less frequently. Consequently, more Kawaguchi grade II and III surgeries were performed in the NCR group (41.6% vs. 30.5% and 14.5% vs. 7.1%, respectively). A higher percentage of surgeries performed in high-volume centers was detected in the NCR group (70.2% vs. 59.0%, *p* < 0.001). The operative time for a CR was 385 min, compared to 300 min (*p* < 0.001) for an NCR, and more patients required intraoperative blood transfusions in the CR group (8.6% vs. 3.0%, *p* < 0.001) with a comparable blood loss (150 mL for both groups, *p* = 0.265). Conversion to laparotomy occurred at a similar rate in both groups (9.1% vs. 9.6%, *p* = 0.760).

Postoperative morbidity was 34.6% and 17.9% in the CR and NCR groups, respectively (*p* < 0.001), with a difference mainly related to a higher incidence of intestinal complications (6.9% vs. 1.4%), superficial site infections (3.2% vs. 0.8%) and abdominal collections (8.1% vs. 2.2%). A statistically significant difference was found specifically in postoperative major complications (CD > 2): 11.6% and 5.4% in the CR and NCR groups (*p* < 0.001), within a similar mortality rate. Hospital stay after CR was significantly longer than after NCR (7 vs. 5 days, *p* < 0.001).

In the univariate logistic regression (Table 3), technical complexity (OR 1.91 for grade III, *p* = 0.005), combined resection (OR 2.30, *p* < 0.001), longer operative time (OR 1.003, *p* < 0.001), higher blood loss (OR 1.0007, *p* < 0.001), intraoperative blood transfusions (OR 2.04, *p* = 0.033) and conversion to laparotomy (OR 2.29, *p* < 0.001) were found to negatively influence the rate of major complications, when surgery performed in high-volume centers is associated with a reduction in major complications (OR 0.57, *p* = 0.001). In the multivariate analysis, technical complexity (OR 1.79 for grade III, *p* = 0.024), combined resection (OR 2.04, *p* = 0.001), operative time (OR 1.002, *p* = 0.021), conversion to laparotomy (OR 1.79, *p* = 0.018) and volume of the center (OR 0.57, *p* = 0.002) remained significant.

### 3.3. Comparison Between CR and NCR Groups After Matching

After matching, the CR and NCR groups consisted of 407 and 822 patients, respectively. The characteristics of patients were comparable (Table 1). While the type of hepatic resection maintained different characteristics between the two groups, technical complexity grades were similar because of the matching process.

In the CR group a longer operative time was confirmed (385 vs. 270 min, *p* < 0.001), as well as a higher need for intraoperative blood transfusions (8.6% vs. 2.7%, *p* < 0.001). A similar rate of conversions to laparotomy was detected. Postoperative complications were higher in the CR group (34.6% vs. 14.8%, *p* < 0.001) as well as major postoperative complications (11.5% vs. 5.2%, *p* < 0.001) and the length of hospital stay (7 vs. 5 days, *p* < 0.001) (Table 2).

In the univariate and multivariate analysis (Table 4), technical complexity (OR 2.05, *p* = 0.045), combined resection (OR 1.86, *p* = 0.011), longer operative time (OR 1.002, *p* = 0.01) and conversion to laparotomy (OR 1.90, *p* = 0.045) were confirmed to be associated with a higher rate of major postoperative complications, while surgery performed at a high-volume center is associated with a reduction in major complications (OR 0.38, *p* < 0.001).

## 4. Discussion

In the present study, a comparison of outcomes of minimally invasive hepatic resections for CRLM versus combined minimally invasive resections of synchronous colorectal tumor and hepatic metastases was performed. Our findings indicate that while morbidity is lower in pure hepatic resections, the combination of the colorectal component increases postoperative morbidity even when using MI approaches. Despite the expanding adoption of minimally invasive techniques, our data suggest that indications for combined resections remain selected, particularly in patients who require more complex colorectal surgery or major liver resections. Therefore, MI does not yet justify broadening indications for combined resection beyond carefully selected patients. Our findings support the recommendations of the Italian Consensus [34] and the multisociety/EAHPBA consensus [18]: combined MI surgery is feasible and beneficial in specific contexts but is not uniformly appropriate. Institutions seeking to adopt combined MI resections should ensure adequate experience, meticulous selection, and multidisciplinary collaboration.

Although there was a progressive increase in the number of patients included each year during the enrollment period, reflecting the growing adoption of minimally invasive techniques in Italy even for more complex procedures, the ratio of combined resection remained stable throughout the study period. This stability reflects careful patient selection and a consistent recruitment policy regarding combined treatment for CRLM.

In this study, we used a cut-off of 10 MILS for CRLM per year to differentiate between high-volume and low-volume centers. This threshold was chosen arbitrarily based on two previous Italian studies, which considered 20 and 24 MILS per year as cut-offs [35,36]. Since our study exclusively focuses on surgeries performed for CRLM, we considered a value of 10 MILS per year as comparable. Further studies are required to establish a precise evidence-based definition of high-volume centers.

Several prior meta-analyses and systematic reviews have addressed the question of simultaneous (combined) versus staged resections in synchronous colorectal liver metastases. For example, “Timing of resection of synchronous colorectal liver metastasis: A systematic review and meta-analysis” [22] included 33 studies with over 6400 patients and found that while simultaneous resection reduced the length of hospital stay, it was associated with a slight increase in perioperative mortality. Similarly, in a recent network meta-analysis [37] (46 studies, ~21,000 patients) comparing simultaneous, colorectal-first, and liver-first strategies, simultaneous resections showed less blood loss and shorter hospital stays, better macroscopic clearance, but no clear improvement in morbidity or survival over other strategies.

Sijberden et al. [16] published a large multicenter retrospective cohort study in which simultaneous resection for synchronous colorectal liver metastases was associated with acceptable outcomes mainly in patients undergoing minor liver resections, whereas major liver resections resulted in higher morbidity and longer hospital stay. Higher ASA grade and major hepatectomy were independent predictors of severe morbidity, supporting the need for careful patient selection when considering a simultaneous approach.

Radomski et al. [7] showed in their article that the minimally invasive CR in a low-risk colorectal and low-risk hepatic procedure has a significantly lower morbidity rate compared to similar open resections but higher compared to isolated minimally invasive colorectal or hepatic procedures. This is consistent with our data and it adds the information that the minimally invasive approach, in addition to being safe, provides an advantage in terms of morbidity compared to the open technique.

Our results confirm that combined MI procedures did not reduce serious morbidity compared to staged or hepatic-only resections; the presence of a colorectal resection added risk, especially when the colorectal resection was complex (e.g., rectal vs. right colon). This supports the view that simultaneous MI resections may be safe in well-selected patients with limited liver disease and lower complexity of the colorectal procedure but are not universally applicable. Intestinal complications, SSI and abdominal infections are the type of complication with more imbalance between the CR and NCR groups and they are possibly linked to the colic resection. Despite the increased risk of postoperative complications, CRs do not increase the conversion to laparotomy rate (9.09% vs. 7.91%, for CRs and NCRs, respectively, *p* = 0.479). Prolonged operative time and the need for more blood transfusions in the CR group represent significant metabolic and physiological stressors—driving catabolism, insulin resistance, and immune suppression—that could have an impact on the postoperative outcome. A factor to be discussed for a possible mitigation strategy of postoperative complications is the centralization of this type of operation: in the present cohort patients who underwent surgery in a high-volume center had an odds ratio for severe complications of 0.37 (0.24–0.58, *p* < 0.001) in the multivariate analysis after matching, highlighting the importance of institutional experience. The centralization of cases in high-volume centers is associated with improved outcomes not only in resections for CRLM but also in hepatic resections performed for other pathologies and even across other surgical specialties [38,39,40,41]. However, the underlying reasons for this advantage have not yet been clearly elucidated. While surgical expertise undoubtedly plays a key role, perioperative management—including prehabilitation, nutritional optimization, and standardized care pathways—may also contribute substantially. Further studies are needed to clarify the relative impact of these components.

The Italian consensus on minimally invasive simultaneous resections for synchronous liver metastasis [34] and primary colorectal cancer provides specific guidance on patient selection, procedural combinations, and technical demands. The consensus emphasizes that minimally invasive simultaneous resection remains promising, but that strong evidence from randomized trials is lacking, and that complexity of the resection (either colorectal or hepatic), patient comorbidities, and institutional expertise should guide decision-making. Our findings reinforce these recommendations: even in centers with MI expertise, morbidity remains higher when the colorectal operation is added, particularly when the colorectal procedure itself is of higher risk or the liver component is large.

In the European guideline/consensus statements [18], terminology, staging, and treatment pathways for synchronous colorectal liver metastases are addressed: one key point is that MI surgery for either or both components (primary and metastasis) is considered acceptable in appropriate settings, but data for fully simultaneous MI surgery are limited. In this consensus, it is acknowledged that morbidity—although improving—is still higher when both operations are performed together, especially in patients needing major resections or when disease burden is large. This agrees with our observation that MI does mitigate but does not eliminate the additional risk imposed by combining colorectal and hepatic resections.

One strength of our work is that it is designed within the I Go MILS group registry, which allows more robust real-world data, with variation in disease burden, surgical complexity, and procedural combinations. Registry data have increasingly been used to assess outcomes of MI liver surgery and combined operations, bridging gaps left by smaller institutional series. These registry-derived insights show that MI techniques are beneficial but that patient selection remains key.

Also in the present study, registry data provide useful insights in combined colorectal and hepatic resections. First, morbidity remains dependent on the colorectal component. Our data suggest that adding a colorectal resection—even when MI—is not neutral; simpler colorectal resections (e.g., right colectomy) are better tolerated in combined settings than more complex procedures (lower rectal resections, multiquadrant resections). Minimally invasive surgery hence mitigates but does not abolish risk. The MI approach reduces some morbidity, shortens hospital stay, and lessens blood loss—as meta-analyses show—but the benefit is less pronounced when both colorectal and hepatic resections are combined, especially if either is complex. Clinical decision-making must remain individualized. Based on our results and those in the literature, combined MI resections should be reserved for patients with favorable profiles: low comorbidity load, limited number/size of liver metastases, less complex colorectal surgery, and when both colorectal and liver surgeries can be performed with MI safely.

Indications for combined surgery have not yet changed. Despite the spread of MI techniques, our data do not support expanding the indications for combined resection beyond what the Italian consensus or EAHPBA and multisociety guidelines currently recommend.

As in all observational and registry-based studies, selection bias remains a concern: patients undergoing combined operations are often those with less extensive disease, with better physiological reserve, and treated in centers with high expertise. Differences in definitions of morbidity (e.g., Clavien–Dindo grading), variation in what is considered “major” hepatic or colorectal resection, and heterogeneity among centers also complicate comparisons. Also, long-term oncologic outcomes and quality of life are less frequently reported in MI simultaneous series, limiting conclusions about equivalence or superiority in survival. The relevance of disease-free survival, overall survival, and time-to-adjuvant therapy in guiding therapeutic decisions underscores the necessity for additional well-designed studies aimed at addressing the challenge of selecting the optimal surgical strategy in the management of synchronous CRLM. Another issue is the lack of more detailed data—within the registry of liver surgery—about colic surgery (e.g., right vs. left colectomy, stoma vs. no stoma), which have an impact on the difficulty of the surgical intervention and can influence the postoperative outcomes. Additionally, a factor that could influence outcomes is the patient’s nutritional status (albumin or prealbumin levels, Subjective Global Assessment scores, CT-derived sarcopenia measures, or longitudinal BMI trends) and the type of prehabilitation program they underwent; unfortunately, it was not possible to include these variables in this study as they are not available in the I Go MILS Registry.

To address remaining uncertainties, the following items need a detailed evaluation: better patient selection criteria and risk stratification tools, possibly integrating tumor biology (genomics), patient comorbidities, and frailty; standardized reporting of morbidity (including perioperative and delayed), conversion rates, hospital stay, functional recovery, and patient-reported outcomes; long-term data on disease-free survival, overall survival, and quality of life comparing MI simultaneous versus staged strategies.

## 5. Conclusions

In summary, our data confirm that while minimally invasive hepatic resections alone show favorable postoperative morbidity, the addition of colorectal surgery for synchronous disease continues to entail higher morbidity even when MI techniques are used. Furthermore, CRs are associated with longer operative times and a higher number of transfusions, but they do not result in an increased rate of conversion to laparotomy. In management of CRLM, accurate preoperative patient selection is a key step in guiding treatment allocation. Further research is needed to know how to establish the best patient for CR, in terms of comorbidities, characteristics of the primary tumor and characteristics of liver metastasis. In case a CR is the desired strategy, performing it in a high-volume center can help to reduce morbidity.

## Figures and Tables

**Table 1:** Demographic and clinical characteristics before and after Nearest Neighbor Matching. Technical complexity based on Kawaguchi et al. [31]. BMI: body mass index. NA: not applicable. Categorical variables were compared using chi-squared test or Fisher’s exact test, as appropriate. Continuous variables were analyzed using the Mann–Whitney U test. In bold, *p*-values < 0.050.

	Before Matching			After Matching		
	Combined n = 407	Not Combined n = 1879	*p*-Value	Combined n = 407	Not Combined n = 822	*p*-Value
**Age** (years) median [25–75%]	67 [58–75]	67 [59–75]	0.527	67 [58–75]	67 [59–75]	0.942
**Sex,** male	238 (58.48%)	1155 (61.47%)	0.262	238 (58.48%)	495 (60.22%)	0.558
**BMI** (kg/m^2^) median [25–75%]	25 [23–28]	25 [23–28]	0.465	25 [23–28]	25 [23–28]	0.709
**Portal hypertension**	0	11 (0.59%)	0.231	0	5 (0.61%)	0.177
**Underlying liver parenchyma**			0.279			0.898
Healthy	318 (78.13%)	1381 (73.50%)		318 (78.13%)	626 (76.16%)	
Steatosis	82 (20.15%)	452 (24.06%)		82 (20.15%)	178 (21.65%)	
Chronic hepatocellular dysfunction	5 (1.23%)	28 (1.49%)		5 (1.23%)	13 (1.58%)	
Cirrhosis	2 (0.49%)	18 (0.96%)		2 (0.49%)	5 (0.61%)	
**Diameter of the largest lesion ≥ 3 cm**	124 (30.47%)	737 (39.22%)	**0.001**	124 (30.47%)	260 (31.63%)	0.679
**Liver first**	NA	276 (14.69%)		NA	122 (14.84%)	
**Resection type**			**<0.001**			**0.005**
Wedge resection	221 (54.30%)	655 (34.86%)		221 (54.30%)	368 (44.77%)	
Anatomical segmentectomy	72 (17.69%)	494 (26.29%)		72 (17.69%)	187 (22.75%)	
Left lateral sectionectomy	33 (8.11%)	170 (9.05%)		33 (8.11%)	105 (12.77%)	
Right anterior sectionectomy	43 (10.57%)	226 (12.03%)		43 (10.57%)	73 (8.88%)	
Right posterior sectionectomy	2 (0.49%)	82 (4.36%)		2 (0.49%)	20 (2.43%)	
Left hepatectomy	17 (4.18%)	100 (5.32%)		17 (4.18%)	30 (3.65%)	
Right hepatectomy	8 (1.97%)	103 (5.48%)		8 (1.97%)	25 (3.04%)	
Right/left trisectionectomy	4 (0.98%)	16 (0.85%)		4 (0.98%)	5 (0.61%)	
Central hepatectomy	1 (0.25%)	9 (0.48%)		1 (0.25%)	3 (0.36%)	
ALPPS	6 (1.47%)	24 (1.28%)		6 (1.47%)	6 (0.73%)	
**Technical Complexity**			**<0.001**			0.263
Grade I	254 (62.41%)	825 (43.91%)		254 (62.41%)	473 (57.54%)	
Grade II	124 (30.47%)	781 (41.56%)		124 (30.47%)	282 (34.31%)	
Grade III	29 (7.13%)	273 (14.53%)		29 (7.13%)	67 (8.15%)	
**Approach**			0.845			0.698
Hybrid	8 (1.97%)	37 (1.97%)		8 (1.97%)	20 (2.43%)	
Laparoscopic	341 (83.78%)	1548 (82.38%)		341 (83.78%)	666 (81.02%)	
Single-port	0	1 (0.05%)		0	1 (0.12%)	
Robotic	58 (14.25%)	293 (15.59%)		58 (14.25%)	135 (16.42%)	
**High-volume center, >10 cases/year**	240 (58.97%)	1320 (70.25%)	**<0.001**	240 (58.97%)	526 (63.99%)	0.087

**Table 2:** Intraoperative and postoperative data before and after Nearest Neighbor Matching. RBC: red blood cells, CD: Clavien–Dindo classification, SSI: superficial site infections. Categorical variables were compared using chi-squared test or Fisher’s exact test, as appropriate. Continuous variables were analyzed using the Mann–Whitney U test. In bold, *p*-values < 0.05.

	Before Matching			After Matching		
	Combined n = 407	Not Combined n = 1879	*p*-Value	Combined n = 407	Not Combined n = 822	*p*-Value
**Operative time** (min) median [25–75%]	385 [290–483]	300 [210–375]	**<0.001**	385 [290–483]	270 [180–360]	**<0.001**
**Blood loss** (mL) median [25–75%]	150 [80–300]	150 [90–300]	0.265	150 [80–300]	150 [50–260]	0.346
**Pringle maneuver**	202 (49.63%)	1244 (66.21%)	**<0.001**	202 (49.63%)	517 (62.90%)	**<0.001**
**Intraoperative RBC transfusion**	35 (8.6%)	57 (3.03%)	**<0.001**	35 (8.6%)	22 (2.68%)	**<0.001**
**Conversion to laparotomy**	37 (9.09%)	180 (9.58%)	0.76	37 (9.09%)	65 (7.91%)	0.479
Hemorrhage	2 (0.49%)	19 (1.01%)		2 (0.49%)	4 (0.49%)	
Adhesions	2 (0.49%)	52 (2.77%)		2 (0.49%)	18 (2.19%)	
Oncologic reasons	28 (6.88%)	94 (5%)		28 (6.88%)	38 (4.62%)	
Iatrogenic injury	0	7 (0.37%)		0	1 (0.12%)	
Anesthesiological reasons	5 (1.23%)	8 (0.43%)		5 (1.23%)	4 (0.49%)	
**Postoperative complications**	141 (34.64%)	337 (17.94%)	**<0.001**	141 (34.64%)	122 (14.84%)	**<0.001**
Bleeding	6 (1.47%)	21 (1.12%)		6 (1.47%)	6 (0.73%)	
Bile leak	11 (2.70%)	48 (2.55%)		11 (2.70%)	20 (2.43%)	
Intestinal complications	28 (6.88%)	26 (1.38%)		28 (6.88%)	11 (1.34%)	
SSI	13 (3.19%)	15 (0.80%)		13 (3.19%)	7 (0.85%)	
Abdominal infections	33 (8.11%)	41 (2.18%)		33 (8.11%)	15 (1.82%)	
Pneumonia	15 (3.69%)	51 (2.71%)		15 (3.69%)	16 (1.95%)	
Liver failure	5 (1.23%)	39 (2.08%)		5 (1.23%)	9 (1.09%)	
**Postoperative major complication** (CD > 2)	47 (11.55%)	101 (5.38%)	**<0.001**	47 (11.55%)	42 (5.11%)	**<0.001**
**Length of hospital stay** (days) median [25–75%]	7 [6–11]	5 [4–6]	**<0.001**	7 [6–11]	5 [4–6]	**<0.001**
**90-day mortality**	1 (0.25%)	8 (0.43%)	1	1 (0.25%)	2 (0.24%)	1

**Table 3:** Univariate and multivariate logistic regression before Nearest Neighbor Matching. RBC: red blood cells, technical complexity based on Kawaguchi et al. [31], OR: odds ratio, CI: confidence interval, BMI: body mass index. In bold, *p*-values < 0.05.

Variable	OR (95% CI)	Univariate Analysis for Predictors of Postoperative Severe Complications (*p*-Value)	OR (95% CI)	Multivariate Analysis for Predictors of Postoperative Severe Complications (*p*-Value)
**Age,** continuous	1.003 (0.988–1.019)	0.676		
**Sex,** male	1.20 (0.86–1.68)	0.282		
**BMI,** continuous	0.99 (0.95–1.03)	0.646		
**Underlying liver parenchyma**				
Healthy	reference			
Steatosis	1.06 (0.72–1.57)	0.752		
Chronic hepatocellular dysfunction	1.47 (0.44–4.90)	0.528		
Cirrhosis	0.77 (0.10–5.85)	0.805		
**Diameter of the largest lesion ≥ 3 cm**	1.01 (0.71–1.42)	0.964		
**Technical complexity**				
Grade I	reference		reference	
Grade II	1.10 (0.76–1.60)	0.615	1.11 (0.75–1.64)	0.614
Grade III	1.91 (1.21–3.00)	**0.005**	1.79 (1.08–2.96)	**0.024**
**Approach**				
Laparoscopic	reference			
Hybrid	2.40 (0.99–5.78)	0.052		
Robotic	1.34 (0.88–2.08)	0.172		
**Approach,** combined surgery	2.30 (1.60–3.31)	**<0.001**	2.04 (1.35–3.08)	**0.001**
**Operative time**, continuous	1.003 (1.002–1.004)	**<0.001**	1.002 (1.000–1.003)	**0.021**
**Blood loss**, continuous	1.0007 (1.0002–1.0011)	**0.004**	1.000 (0.999–1.001)	0.622
**Pringle maneuver**	1.19 (0.83–1.69)	0.343		
**Intraoperative RBC transfusion**	2.04 (1.06–3.92)	**0.033**	0.89 (0.41–1.91)	0.759
**Conversion to laparotomy**	2.29 (1.47–3.56)	**<0.001**	1.79 (1.11–2.90)	**0.018**
**High-volume center,** >10 cases/year	0.57 (0.41–0.80)	**0.001**	0.57 (0.40–0.81)	**0.002**

**Table 4:** Univariate and multivariate logistic regression after Nearest Neighbor Matching. RBC: red blood cells, technical complexity based on Kawaguchi et al. [31], OR: odds ratio, CI: confidence interval, BMI: body mass index. In bold, *p*-values < 0.05.

Variable	OR (95% CI)	Univariate Analysis for Predictors of Postoperative Severe Complications (*p*-Value)	OR (95% CI)	Multivariate Analysis for Predictors of Postoperative Severe Complications (*p*-Value)
**Age,** continuous	1.008 (0.989–1.028)	0.411		
**Sex,** male	0.91 (0.58–1.41)	0.667		
**BMI,** continuous	1.04 (0.99–1.07)	0.134		
**Underlying liver parenchyma**				
Healthy	reference			
Steatosis	1.09 (0.65–1.83)	0.743		
Chronic hepatocellular dysfunction	0.77 (0.10–5.87)	0.801		
Cirrhosis	2.18 (0.26–18.39)	0.473		
**Diameter of the largest lesion ≥ 3 cm**	1.13 (0.71–1.78)	0.603		
**Technical Complexity**				
Grade I	reference		reference	
Grade II	1.36 (0.85–2.19)	0.203	1.27 (0.77–2.10)	0.347
Grade III	2.72 (1.42–5.18)	**0.002**	2.12 (1.05–4.31)	**0.037**
**Approach**				
Laparoscopic	reference			
Hybrid	2.34 (0.79–6.93)	0.126		
Robotic	1.44 (0.84–2.49)	0.187		
**Approach,** combined surgery	2.42 (1.57–3.74)	**<0.001**	1.92 (1.18–3.11)	**0.008**
**Operative time**, continuous	1.003 (1.002–1.005)	**<0.001**	1.002 (1.001–1.004)	**0.011**
**Blood loss**, continuous	1.001 (0.999–1.002)	0.092		
**Pringle maneuver**	1.51 (0.95–2.39)	0.078		
**Intraoperative RBC transfusion**	2.20 (1.01–4.80)	**0.048**	0.94 (0.40–2.17)	0.878
**Conversion to laparotomy**	2.69 (1.50–4.82)	**0.001**	1.92 (1.02–3.63)	**0.044**
**High-volume center,** >10 cases/year	0.38 (0.25–0.59)	**<0.001**	0.37 (0.24–0.58)	**<0.001**

## Data Availability

The data presented in this study are available on request from the corresponding author due to privacy reasons.

## Abbreviations

The following abbreviations are used in this manuscript:

CRLM	Colorectal Liver Metastases
CRs	Combined Resections
NCRs	Non-Combined Resections
MILR	Minimally Invasive Liver Resection
OR	Odds Ratio
CI	Confidence Interval
CD	Clavien–Dindo classification
SSI	Superficial Site Infection
RBC	Red Blood Cells
BMI	Body Mass Index
MI	Minimally Invasive

## References

[1] Keum N.N., Giovannucci E. (2019). Global Burden of Colorectal Cancer: Emerging Trends, Risk Factors and Prevention Strategies. Nat. Rev. Gastroenterol. Hepatol..

[2] Sung H., Ferlay J., Siegel R.L., Laversanne M., Soerjomataram I., Jemal A., Bray F. (2021). Global Cancer Statistics 2020: GLOBOCAN Estimates of Incidence and Mortality Worldwide for 36 Cancers in 185 Countries. CA Cancer J. Clin..

[3] Cervantes A., Adam R., Roselló S., Arnold D., Normanno N., Taïeb J., Seligmann J., De Baere T., Osterlund P., Yoshino T. (2023). Metastatic Colorectal Cancer: ESMO Clinical Practice Guideline for Diagnosis, Treatment and Follow-up. Ann. Oncol..

[4] Brouquet A., Mortenson M.M., Vauthey J.N., Rodriguez-Bigas M.A., Overman M.J., Chang G.J., Kopetz S., Garrett C., Curley S.A., Abdalla E.K. (2010). Surgical Strategies for Synchronous Colorectal Liver Metastases in 156 Consecutive Patients: Classic, Combined or Reverse Strategy?. J. Am. Coll. Surg..

[5] Boudjema K., Locher C., Sabbagh C., Ortega-Deballon P., Heyd B., Bachellier P., Métairie S., Paye F., Bourlier P., Adam R. (2021). Simultaneous versus Delayed Resection for Initially Resectable Synchronous Colorectal Cancer Liver Metastases: A Prospective, Open-Label, Randomized, Controlled Trial. Ann. Surg..

[6] Frühling P., Strömberg C., Isaksson B., Urdzik J. (2023). A Comparison of the Simultaneous, Liver-First, and Colorectal-First Strategies for Surgical Treatment of Synchronous Colorectal Liver Metastases at Two Major Liver-Surgery Institutions in Sweden. HPB.

[7] Radomski S.N., Chen S.Y., Stem M., Done J.Z., Efron J.E., Safar B., Atallah C. (2023). Simultaneous Resection of Colorectal Cancer and Synchronous Colorectal Liver Metastases: A Risk Stratified Analysis of the NSQIP Database. J. Surg. Oncol..

[8] Giuliante F., Viganò L., De Rose A.M., Mirza D.F., Lapointe R., Kaiser G., Barroso E., Ferrero A., Isoniemi H., Lopez-Ben S. (2021). Liver-First Approach for Synchronous Colorectal Metastases: Analysis of 7360 Patients from the LiverMetSurvey Registry. Ann. Surg. Oncol..

[9] Hernandez Dominguez O., Yilmaz S., Steele S.R. (2023). Stage IV Colorectal Cancer Management and Treatment. J. Clin. Med..

[10] Emile S.H., Horesh N., Garoufalia Z., Gefen R., Zhou P., Wexner S.D. (2024). Propensity-Score Matched Outcomes of Resection of Stage IV Primary Colon Cancer with and without Simultaneous Resection of Liver Metastases. Updates Surg..

[11] Wang S.H., Song L., Tang J.Y., Sun W.-P., Li Z. (2022). Safety and Long-Term Prognosis of Simultaneous versus Staged Resection in Synchronous Colorectal Cancer with Liver Metastasis: A Systematic Review and Meta-Analysis. Eur. J. Med. Res..

[12] Adam R., de Gramont A., Figueras J., Kokudo N., Kunstlinger F., Loyer E., Poston G., Rougier P., Rubbia-Brandt L., Sobrero A. (2015). Managing Synchronous Liver Metastases from Colorectal Cancer: A Multidisciplinary International Consensus. Cancer Treat. Rev..

[13] Mayo S.C., Pulitano C., Marques H., Lamelas J., Wolfgang C.L., De Saussure W., Choti M.A., Gindrat I., Aldrighetti L., Barrosso E. (2013). Surgical Management of Patients with Synchronous Colorectal Liver Metastasis: A Multicenter International Analysis. J. Am. Coll. Surg..

[14] Pinto F., Di Pangrazio M., Martinino A., Todeschini L., Toti F., Cristin L., Caimano M., Mattia A., Bianco G., Spoletini G. (2024). Laparoscopic versus Open Liver Resection for Colorectal Liver Metastasis: An Umbrella Review. Front. Oncol..

[15] Milazzo M., Todeschini L., Caimano M., Mattia A., Cristin L., Martinino A., Bianco G., Spoletini G., Giovinazzo F. (2024). Surgical Resection in Colorectal Liver Metastasis: An Umbrella Review. Cancers.

[16] Sijberden J.P., Zimmitti G., Conci S., Russolillo N., Masetti M., Cipriani F., Lanari J., Görgec B., Benedetti Cacciaguerra A., Rotellar F. (2023). Simultaneous Resection of Colorectal Cancer and Synchronous Liver Metastases: What Determines the Risk of Unfavorable Outcomes? An International Multicenter Retrospective Cohort Study. Int. J. Surg..

[17] Ratti F., Serenari M., Zanello M., Fuks D., Rottoli M., Masetti M., Tribillon E., Ravaioli M., Elmore U., Rosati R. (2021). Team Strategy Optimization in Combined Resections for Synchronous Colorectal Liver Metastases. A Comparative Study with Bootstrapping Analysis. World J. Surg..

[18] Siriwardena A.K., Serrablo A., Fretland Å.A., Wigmore S.J., Ramia-Angel J.M., Malik H.Z., Stättner S., Søreide K., Zmora O., Meijerink M. (2023). The Multi-Societal European Consensus on the Terminology, Diagnosis and Management of Patients with Synchronous Colorectal Cancer and Liver Metastases: An E-AHPBA Consensus in Partnership with ESSO, ESCP, ESGAR, and CIRSE. HPB.

[19] Carrion-Alvarez L., Primavesi F., Søreide K., Sochorova D., Diaz-Nieto R., Dopazo C., Serrablo A., Edhemovic I., Stättner S. (2025). Liver Metastases from Colorectal Cancer: A Joint ESSO–EAHPBA–UEMS Core Curriculum Collaboration. Eur. J. Surg. Oncol..

[20] Reddy S.K., Pawlik T.M., Zorzi D., Gleisner A.L., Ribero D., Assumpcao L., Barbas A.S., Abdalla E.K., Choti M.A., Vauthey J.N. (2007). Simultaneous Resections of Colorectal Cancer and Synchronous Liver Metastases: A Multi-Institutional Analysis. Ann. Surg. Oncol..

[21] Snyder R.A., Hao S., Irish W., Zervos E.E., Tuttle-Newhall J.E., Parikh A.A. (2020). Thirty-Day Morbidity after Simultaneous Resection of Colorectal Cancer and Colorectal Liver Metastasis: American College of Surgeons NSQIP Analysis. J. Am. Coll. Surg..

[22] Gumiero J.L., de Oliveira B.M.S., de Neto O.P.A., Pandini R.V., Gerbasi L.S., Figueiredo M.N., Kruger J.A.P., Seid V.E., Araujo S.E.A., Tustumi F. (2022). Timing of Resection of Synchronous Colorectal Liver Metastasis: A Systematic Review and Meta-Analysis. J. Surg. Oncol..

[23] Ratti F., Fiorentini G., Cipriani F., Catena M., Paganelli M., Aldrighetti L. (2018). Laparoscopic vs Open Surgery for Colorectal Liver Metastases. JAMA Surg..

[24] Fretland A.A., Dagenborg V.J., Bjørnelv G.M.W., Kazaryan A.M., Kristiansen R., Fagerland M.W., Hausken J., Tønnessen T.I., Abildgaard A., Barkhatov L. (2018). Laparoscopic Versus Open Resection for Colorectal Liver Metastases: The OSLO-COMET Randomized Controlled Trial. Ann. Surg..

[25] Robles-Campos R., Lopez-Lopez V., Brusadin R., Lopez-Conesa A., Gil-Vazquez P.J., Navarro-Barrios Á., Parrilla P. (2019). Open versus Minimally Invasive Liver Surgery for Colorectal Liver Metastases (LapOpHuva): A Prospective Randomized Controlled Trial. Surg. Endosc..

[26] Masetti M., Fallani G., Ratti F., Ferrero A., Giuliante F., Cillo U., Guglielmi A., Ettorre G.M., Torzilli G., Vincenti L. (2022). Minimally Invasive Treatment of Colorectal Liver Metastases: Does Robotic Surgery Provide Any Technical Advantages over Laparoscopy? A Multicenter Analysis from the IGoMILS (Italian Group of Minimally Invasive Liver Surgery) Registry. Updates Surg..

[27] Ratti F., Cipriani F., Fiorentini G., Burgio V., Ronzoni M., Della Corte A., Cascinu S., De Cobelli F., Aldrighetti L. (2021). Evolution of Surgical Treatment of Colorectal Liver Metastases in the Real World: Single Center Experience in 1212 Cases. Cancers.

[28] Ratti F., Ferrero A., Guglielmi A., Cillo U., Giuliante F., Mazzaferro V., De Carlis L., Ettorre G.M., Gruttadauria S., Di Benedetto F. (2023). Ten Years of Italian Mini-Invasiveness: The I Go MILS Registry as a Tool of Dissemination, Characterization and Networking. Updates Surg..

[29] Aldrighetti L., Ratti F., Cillo U., Ferrero A., Ettorre G.M., Guglielmi A., Giuliante F., Calise F. (2017). Diffusion, Outcomes and Implementation of Minimally Invasive Liver Surgery: A Snapshot from the I Go MILS (Italian Group of Minimally Invasive Liver Surgery) Registry. Updates Surg..

[30] Dindo D., Demartines N., Clavien P.A. (2004). Classification of Surgical Complications: A New Proposal with Evaluation in a Cohort of 6336 Patients and Results of a Survey. Ann. Surg..

[31] Kawaguchi Y., Fuks D., Kokudo N., Gayet B. (2018). Difficulty of Laparoscopic Liver Resection. Ann. Surg..

[32] Austin P.C. (2011). An Introduction to Propensity Score Methods for Reducing the Effects of Confounding in Observational Studies. Multivar. Behav. Res..

[33] Heinz P., Wendel-Garcia P.D., Held U. (2024). Impact of the Matching Algorithm on the Treatment Effect Estimate: A Neutral Comparison Study. Biom. J..

[34] Rocca A., Cipriani F., Belli G., Berti S., Boggi U., Bottino V., Cillo U., Cescon M., Cimino M., Corcione F. (2021). The Italian Consensus on Minimally Invasive Simultaneous Resections for Synchronous Liver Metastasis and Primary Colorectal Cancer: A Delphi Methodology. Updates Surg..

[35] Viganò L., Cimino M., Aldrighetti L., Ferrero A., Cillo U., Guglielmi A., Ettorre G.M., Giuliante F., Dalla Valle R., Mazzaferro V. (2020). Multicentre Evaluation of Case Volume in Minimally Invasive Hepatectomy. Br. J. Surg..

[36] Torzilli G., Viganò L., Giuliante F., Pinna A.D. (2016). Liver Surgery in Italy. Criteria to Identify the Hospital Units and the Tertiary Referral Centers Entitled to Perform It. Updates Surg..

[37] Sijberden J.P., Alvarez Escribano M.S., Kasai M., Ferretti C., Cesaro P., Bnà C., Zaniboni A., Siriwardena A.K., Tanis P.J., Abu Hilal M. (2025). Perioperative Safety and Oncological Efficacy of Simultaneous versus Colorectal and Liver First Two-Staged Resections in Patients with Synchronous Colorectal Liver Metastases: A Systematic Review and Network Meta-Analysis. HPB.

[38] Hata T., Motoi F., Ishida M., Naitoh T., Katayose Y., Egawa S., Unno M. (2016). Effect of Hospital Volume on Surgical Outcomes After Pancreaticoduodenectomy: A Systematic Review and Meta-Analysis. Ann. Surg..

[39] Di J., Lu X.S., Sun M., Zhao Z.M., Zhang C.D. (2024). Hospital Volume-Mortality Association after Esophagectomy for Cancer: A Systematic Review and Meta-Analysis. Int. J. Surg..

[40] Koh Y.X., Zhao Y., Tan I.E.H., Tan H.L., Chua D.W., Loh W.L., Tan E.K., Teo J.Y., Au M.K.H., Goh B.K.P. (2024). The Impact of Hospital Volume on Liver Resection: A Systematic Review and Bayesian Network Meta-Analysis. Surgery.

[41] Luo Q., Wang Y., Zhang X. (2025). Association between Hospital Volume and Outcomes in Ovarian Cancer: A Systematic Review. Front. Surg..

